# Routine use of *Staphylococcus aureus *rapid diagnostic test in patients with suspected ventilator-associated pneumonia

**DOI:** 10.1186/cc12849

**Published:** 2013-08-06

**Authors:** Marc Leone, François Malavieille, Laurent Papazian, Bertrand Meyssignac, Nadim Cassir, Julien Textoris, François Antonini, Bernard La Scola, Claude Martin, Bernard Allaouchiche, Sami Hraiech

**Affiliations:** 1 Réanimations du pôle AUR, Hôpital Nord, Assistance Publique - Hôpitaux de Marseille, Aix Marseille Université, Chemin des Bourrely, 13015 Marseille, France; 2 Réanimation, Hôpital Edouard Herriot, Hospices Civils de Lyon, Université Claude Bernard-Lyon 1, place d'Arsonval, 69437 Lyon cedex 03, France; 3 UMR CNRS 7278, Université Aix Marseille, Faculté de Médecine la Timone, Bld Jean Moulin, 13005 Marseille, France; 4 Service d'anesthésie et de réanimation, Hôpital Nord, Chemin des Bourrely, 13915 Marseille, France

**Keywords:** pneumonia, point-of-care, diagnostic, staphylococcus, resistance

## Abstract

**Introduction:**

In patients with ventilator-associated pneumonia (VAP), administration of an appropriate empirical antimicrobial treatment is associated with improved outcomes, leading to the prescription of broad-spectrum antibiotics, including a drug active against methicillin resistant *Staphylococcus aureus *(MRSA). In order to avoid the overuse of antibiotics, the present study aimed to evaluate the technical characteristics of a rapid diagnostic test (Cepheid Xpert assay) in patients with suspected VAP.

**Methods:**

From June 2011 to June 2012, in patients with suspected VAP, a sample from the bronchialalveolar lavage (BAL) or miniBAL was tested in a point-of-care laboratory for a rapid diagnostic test of methicillin susceptible *Staphylococcus aureus *(MSSA) and MRSA. Then, the result was compared to the quantitative culture with a threshold at 10^4 ^colony-forming units per milliliter for bronchoalveolar lavage and 10^3 ^colony-forming units per milliliter for minibronchoalveolar lavage. The study was performed in three intensive care units at two institutions.

**Results:**

Four hundred, twenty-two samples from 328 patients were analyzed. The culture of 6 (1.1%) and 28 (6.5%) samples were positive for MRSA and MSSA. The test was not interpretable in 41 (9.3%) patients. The negative predictive values of the rapid detection test were 99.7% (98.1 to 99.9%) and 99.8% (98.7 to 99.9%) for MSSA and MRSA, respectively.

**Conclusion:**

The rapid diagnostic test is reliable in excluding the presence of MSSA and MRSA in the samples of patients with suspected VAP. Its utility should be regarded depending on the prevalence of MRSA.

## Introduction

Because survival is improved in the patients receiving appropriate empirical antibiotics, guidelines recommend the coverage of all potential pathogens responsible for an episode of ventilator-associated pneumonia (VAP) [1,2]. Collection of blood and bronchial specimens precedes the administration of empirical antibiotics. The choice of antibiotics is based on the presence of specific risk factors [2]. After the responsible bacteria in samples were identified, guidelines recommend reassessing the antibiotic treatment [2].

Although safe, this strategy exposes the patient to an overuse of broad-spectrum antibiotics [3]. Overuse of antibiotics results in an increase in multidrug resistant pathogens, treatment-related side effects and increased cost of hospitalization. Use of biomarkers, for instance, procalcitonin, failed to be effective in septic ICU patients in deciding whether or not to start antibiotics [4]. The Gram stain of bronchial sputum is not safe enough to use in deciding whether or not to start an antimicrobial treatment [5]. With regard to methicillin-resistant *Staphylococcus aureus *(MRSA), this strategy leads to a wide use of vancomycin or linezolid [6]. These antibiotics are associated with side effects and increased costs [6].

A rapid detection of resistant bacteria can avoid the use of unnecessary antibiotics [7]. Because VAP due to MRSA has been associated with increased mortality [8], the detection test should have an excellent negative predictive value. New diagnostic tests using real time polymerase chain reaction (RT-PCR) detect pathogens in approximately 60 minutes [9]. They can detect both methicillin-susceptible *Staphylococcus aureus *(MSSA) and MRSA in blood, nasal and surgical site secretions. To date, these tests have been poorly assessed in bronchial secretions of patients with suspected VAP.

We hypothesized that the routine use of a rapid detection (rPCR) test in bronchial secretion samples would limit the use of anti-MRSA antibiotics. The performance of the rPCR test has been evaluated on 135 lower respiratory tract secretions. However, only the patients with endotracheal aspirates showing Gram-positive cocci were included in this study [10]. Using the rPCR tests designed for nasal secretions and surgical site fluid, our goal was to assess the technical reliability of the rPCR test for *Staphylococcus aureus *and its concordance with bronchoalveolar (BAL) and miniBAL microbiological result.

## Materials and methods

The study was conducted in three ICUs from two institutions (Aix Marseille Université and Claude Bernard Université Lyon 1). As it was an observational retrospective study, according to French legislation (articles L.1121-1 paragraph 1 and R1121-2, Public Health Code), neither informed consent nor approval of the ethics committee was needed to use data for an epidemiologic study. From June 2011 to June 2012, the patients were selected if a VAP episode was suspected [1].

### Culture and identification of MRSA

The bronchial secretions were collected using a BAL or a mini-BAL. Qualitative and quantitative cultures were performed. A quantitative BAL or mini-BAL was defined as negative if there was less than 10^4 ^colony-forming units (cfu)/mL and 10^3 ^cfu/mL of growth from the culture [11,12].

All respiratory tract samples were divided into two aliquots. A first aliquot was inoculated on Columbia 5% sheep-blood, chocolate PolyViteX and MacConkey agar media (bioMérieux, Marcy l'Etoile, France). Incubation was performed at 36°C under 5% CO_2 _atmosphere for Columbia 5% sheep-blood and chocolate PolyViteX. Growing microorganisms were identified by using matrix-assisted laser desorption ionization time-of-flight [13]. Methicillin resistance was determined for *Staphylococcus aureus *by using a disk-diffusion method (Mast Diagnostics, Amiens, France) after 18 to 24 hours of incubation on Mueller-Hinton agar media (bioMerieux) according with the European Committee on Antimicrobial Susceptibility Testing (EUCAST) breakpoints.

The second aliquot was used to determine the presence or not of *S. aureus *using the rapid detection test. No antibiotic active against MRSA was used before a BAL or mini-BAL was performed. Results were available via the intranet website of each institution.

### rPCR test for *S. aureus*

*Staphylococcus aureus *was identified using a rapid diagnostic test on a platform *Cepheid Xpert real time PCR assay *(Cepheid, Sunnyvale, CA, USA). In the Marseille study center, an Xpert SA Nasal Complete Kit was used. In the Lyon study center, an Xpert MRSA/SA SSTI was used. The extraction, amplification and detection steps take place in different chambers of a self-contained, single-use cartridge containing all reagents required for the bacterial target detection. Samples were adsorbed onto a swab, which was inserted in the extraction buffer vial of the Xpert assay, transferred into the cartridge, and treated according to the manufacturer's instructions. Both the Xpert SA Nasal Complete and the Xpert MRSA/SA SSTI target the staphylococcal protein A (*spa*) gene, the gene for methicillin resistance (*mecA*) and the staphylococcal cassette chromosome (SCC*mec*) inserted into the *S. aureus *chromosomal *attB *insertion site, and an internal-control sample processing control (SPC) three genetic markers (*Bacillus globigii*).

The rPCR test was available 24 h a day and 7 days a week. In Marseille, a microbiologist was responsible for the procedure in a point-of-care laboratory. In Lyon, a clinical research assistant performed the rPCR test directly in the intensive care unit. The detection of a microorganism is accompanied by a positive signal. This detection is possible in 58 minutes.

### Collection of data

We collected the simplified acute physiology score (SAPS 2) at admission, reason for admission and the duration of the ICU stay, day of occurrence of the episode of VAP, result of rPCR test, result of Gram stain and result of microbiological culture. The analysis was conducted in the entire cohort and then in the patients with risk factors for MRSA infection, as defined elsewhere [2].

### Economic assessment

The economic assessment conducted examined rPCR test kit testing as an adjunct for antibiotic management of VAP empirical treatment in 2013 Euros. Estimates of the cost of running a rapid detection test are approximately €45 per test. We considered two approaches to model antibiotic costs in the empirical treatment of VAP: 1) a less expensive option, such as what might be used for patients without renal failure (€50/day), and 2) a more expensive option, such as what might be used for patients with renal impairment (€150/day). The estimate of the duration of empirical antibiotics (three-days) is derived from our prior publications [14].

### Statistical analysis

A statistical analysis was performed using R-project 2.14 for GNU Linux Ubuntu. With respect to continuous variables, data were expressed as median and interquartile range (25% to 75%). With respect to dichotomous variables, percentages were calculated. Regarding MSSA and MRSA, sensitivity, specificity, positive predictive value, negative predictive value, positive likelihood and negative likelihood were computed. In order to assess the efficiency of the rapid diagnostic test, the Youden index was calculated as follows: Youden index = (sensitivity + specificity - 1). A level of *P *below 0.05 was considered as significant.

## Results

Within the study period, 422 samples were analyzed using the rPCR test in 328 patients. The population consisted of 151 (46%) medical patients, 102 (31%) surgical patients and 75 (23%) trauma patients. The SAPS 2 was 39 (27 to 52). The duration of ICU stay was 16 (8 to 36) days. The results of culture were reported as sterile and commensal flora in 168 (40%) and 70 (16%) cases, respectively. At least one microorganism growth considered as significant was observed in 184 samples. *Pseudomonas aeruginosa *was the most frequently isolated species (*n *= 45 (11%)). *S. aureus *was identified in 34 (8.1%) samples, including 28 (6.5%) MSSA and six (1.4%) MRSA.

All patients with MRSA VAP had significant risk factors (Table 1). The results of 44 (10.4%) rPCR tests were given as not interpretable. Forty-one (93%) inconclusive tests were reported in Marseille. The technical characteristics of the rPCR test for MSSA and MRSA identification are reported in Table 2. The negative predictive values, that is, the proportion of subjects with a negative test result who were correctly diagnosed, of the rPCR test were 99.7% (98.1 to 99.9%) and 99.8% (98.7 to 99.9%) for MSSA and MRSA, respectively. Gram stain served to identify Gram positive cocci in 94 (22%) samples. This included 18 (65%) out of 28 positive cultures for MSSA, and 3 (50%) out of 6 positive cultures for MRSA. With respect to MRSA, its sensitivity was below 5%. Its specificity was at 99%. Inconclusive tests were either excluded or included positive tests or negative tests, respectively.

**Table 1:** Risk factors for carrying methicillin-resistant *Staphylococcus aureus*

	Prior duration of hospitalization > 5 days	Antibiotics in the preceding 90 days	Immuno-suppression	Life in medical institution	Chronic hemo- dialysis	Long term catheterization
**1**	1	0	0	0	0	1
**2**	0	1	0	0	0	0
**3**	1	1	1	1	0	0
**4**	1	0	0	0	0	0
**5**	1	1	1	1	0	1
**6**	1	1	1	0	0	1

The rapid diagnostic test was negative for patient n°6.

**Table 2:** Technical features of the rapid PCR test

All patients	Se (%) [95% CI]	Sp (%) [95% CI]	PPV (%) [95% CI]	NPV (%) [95% CI]	LR +	LR -	Youden index
*MSSA* *(Rapid PCR)*	95.8 [78.9 to 99.9]	83.2 [78.9 to 86.9]	27.7 [18.4 to 38.6]	99.7 [98.1 to 99.9]	5.7	0.05	0.79

MSSA* (Rapid PCR)	96.3 [81.0 to 99.9]	75.2 [70.6 to 79.4]	21.0 [14.2 to 29.2]	99.7 [98.1 to 99.9]	3.9	0.05	0.72

*MSSA* *(Rapid PCR)*	85.2 [66.3 to 95.8]	84.8 [80.9 to 88.2]	27.1 [18.5 to 38.6]	98.8 [97.0 to 99.7]	5.6	0.18	0.70

*MSSA (Gram stain)*	62.9 [42.4 to 80.6]	80.7 [76.5 to 84.5]	18.3 [11.0 to 27.7]	97.0 [94.5 to 98.5]	3.3	0.5	0.44

*MRSA* *(Rapid PCR)*	80.0 [28.4 to 99.5]	99.5 [98.3 to 99.9]	66.7 [22.3 to 95.7]	99.8 [98.7 to 99.9]	166.8	0.2	0.79

MRSA* (Rapid PCR)	100.0 [47.8 to 100.0]	71.5 [66.9 to 75.8]	4.0 [1.3 to 9.2]	100.0 [98.7 to 100.0]	3.5	0.00	0.72

*MRSA* *(Rapid PCR)*	100.0 [47.8 to 100.0]	80.3 [77.2 to 84.9]	6.0 [2.0 to 13.5]	100.0 [98.9 to 100.0]	5.3	0.00	0.81

*MRSA* *(Gram stain)*	40.0 [5.3 to 85.3]	78.2 [73.9 to 82.1]	2.2 [0.3 to 7.6]	99.1 [97.4 to 99.8]	1.8	0.8	0.18

**≥ One risk factor**	**Se (%)**	**Sp (%)**	**PPV (%)**	**NPV (%)**	**LR +**	**LR -**	**Youden index**

*MRSA*	80 [28.4 to 99.5]	99.3 [97.6 to 99.9]	66.7 [22.3 to 95.7]	99.7 [98.1 to 99.9]	118.4	0.2	0.79

*Including inconclusive tests as positive; Including inconclusive test as negative

Technical characteristics of the rapid PCR test and Gram stain performed on bronchoalveolar lavage samples in all patients and those with at least one risk factor for methicillin resistant *Staphylococcus aureus *infection

LR, likelihood ratio; MRSA, methicillin-resistant *Staphylococcus aureus; *MSSA, methicillin-susceptible *Staphylococcus aureus*; NPV, negative predictive value; PPV, predictive positive value; Se, sensitivity; Sp, specificity

We identified a specific population of 301 patients with at least one risk factor for MRSA. Prior duration of hospitalization (> 5 days), antibiotic treatment in the preceding 90 days, chronic hemodialysis and central line or implantable device were found in 257, 131, 11 and 149 patients, respectively. The median number of risk factors was two (one to three). In those patients, using only the positive sample for MRSA investigation, the predictive negative value of the rPCR test for MRSA detection was 99.7% (98.1 to 99.9%) (Table 2).

**Figure 1:**
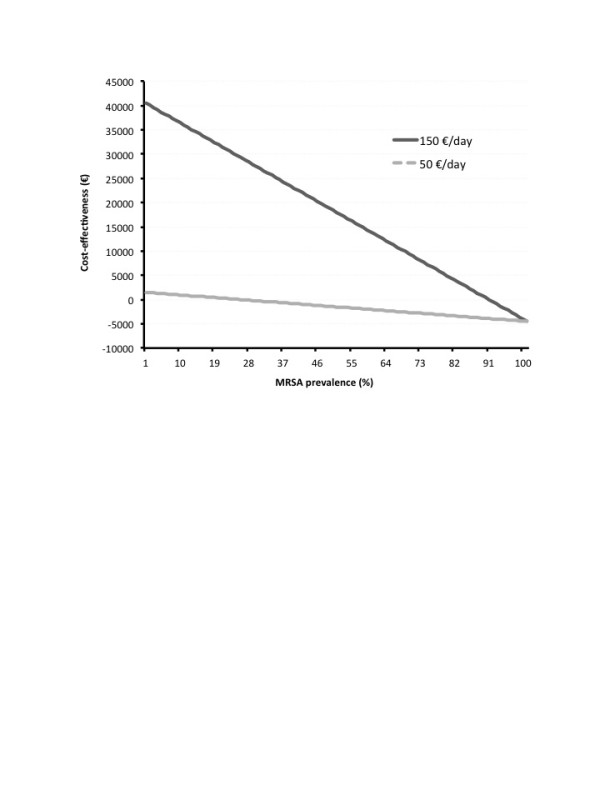
**Cost-effectiveness of the rapid detection test for methicillin-resistant *Staphylococcus aureus *(MRSA) in the bronchial samples of patients with suspected ventilator-associated pneumonia**. The x-axis represents the prevalence of MRSA. The y-axis represents the change in total cost (antimicrobial therapy and rapid diagnostic test) associated with the use of the test (in Euros). Cost is based on consecutive use in 100 patients.

Figure 1 reports the cost-effectiveness analysis depending on the prevalence of MRSA. The cost-effectiveness was based on a strategy including a three-day empirical antimicrobial therapy. Based on a treatment cost at €150/day, the rPCR test was cost-effective, independent of the prevalence of MRSA. Based on a treatment cost at €50/day, the rPCR test was cost-effective for MRSA prevalence below 25%.

## Discussion

Our study shows that the rPCR test is reliable for the detection of *S. aureus *in bronchial secretions of patients with tracheal intubation. The excellent negative predictive value suggests that antibiotics directed against MRSA may not be used in most patients with a negative test. This finding is in agreement with that published in previous studies [10,15]. Importantly, the rPCR test cannot confirm the presence or absence of VAP. The diagnosis of VAP is based on clinical, radiological and microbiological features.

The striking finding of our study is a prevalence rate of MRSA below 2%. This prevalence is lower than that reported in previous studies [14,15]. This is in line with data regarding the decreased number of bacteremias due to MRSA in Europe [16]. This low prevalence is a major limitation of the present study. Indeed, the predictive values of a test depend on the prevalence of the event in the patients being tested. The predictive value of a negative test decreases as soon as the prevalence of the event increases. Then, future studies are required to test the possibility of excluding MRSA in an ICU patient with a clinical suspicion of VAP using a rPCR test when the prevalence of MRSA is high.

In routine, Gram stain is the first microbiological result available for the clinician. Its role remains a matter of debate. A meta-analysis showed that Gram stain is not reliable, with the exception of negative findings [17]. Gram stain may be used to screen the patients at high-risk of MRSA. In a prior study, the rapid diagnostic test was conducted in endotracheal aspirates showing Gram positive cocci in clusters [10]. This pre-screening improved the performance of the rPCR test. However, three out of our six patients with a positive culture for MRSA had a positive Gram stain. Thus, this strategy shows that the number of patients with MRSA is underestimated.

In routine, a diagnostic test should have an excellent reliability [18]. In our series, the rPCR test was inconclusive in around 10% of the samples. This result differs from a study showing that all tests were valid [15]. In our study, this is a limitation of the use of the rPCR test. The test is not interpretable when the DNA cannot be amplified. In practice, this is probably related to the features of bronchial secretions. The lack of fluidity of samples can preclude their analysis by the device. A pre-treatment aimed at increasing the sample fluidity may increase the number of valid tests [15]. Based on local decision, a different rPCR test was used in each of our two centers. One should note that most of the inconclusive tests corresponded to the complete nasal kit. Future studies are required to explore whether the SSTI kit results in less technical failure than the nasal kit.

The cost-effectiveness of the rPCR test is related to the prevalence of MRSA. The estimated cost of the rapid diagnostic test is around €45. Using an expensive treatment option, the routine use of the test remains cost-effective whatever the MRSA prevalence. In contrast, using a less expensive option, the test seems to be less cost-effective above 25% MRSA prevalence. Our results show that risk factors were identified in the six patients with positive cultures for MRSA. Thus, a careful screening of patients at high-risk of MRSA carriage improves the effectiveness of the diagnosis process.

## Conclusion

In unselected patients with suspected VAP, the rPCR test has an excellent negative predictive value. Its routine use should be discussed according to the prevalence of MRSA. In our opinion, this test should be used only in the patients at high-risk of MRSA infection or in endemic situations.

## Key messages

• The prevalence of positive culture for methicillin-resistant *Staphylococcus aureus *is less than 2% in the lower respiratory tract secretions of patients with suspected ventilator-associated pneumonia.

• The negative predictive value of a rapid diagnostic test aiming at identifying *Staphylococcus aureus *in bronchial secretions is excellent.

• The negative predictive value of a rapid diagnostic test aiming at identifying methicillin-resistant *Staphylococcus aureus *in bronchial secretions is excellent.

• The use of a rapid diagnostic test may be associated with a reduced use of antibiotics.

• The cost effectiveness of the rapid diagnostic test should be evaluated according to the prevalence of methicillin-resistant *Stahylococcus aureus*.

## Abbreviations

BAL: bronchial alveolar lavage; CFU: colonies forming unit; EUCAST: European Committee on Antimicrobial Susceptibility Testing; MRSA: methicillin resistant *Staphylococcus aureus*; MSSA: methicillin susceptible *Staphylococcus aureus; *rPCR: rapid detection; RT-PCR: real time polymerase chain reaction; SAPS: simplified acute physiology score; SPC: sample processing control; VAP: ventilator-associated pneumonia.

## Competing interests

The authors declare that they have no competing interests.

## Authors' contributions

ML, CM, BA and LP were involved in the conception of the study. FM, SH, FA and BM participated to the acquisition of data, and ML, LP, BA, FA and JT participated to the interpretation of results. ML, BA, LP, BLS and NC were involved in drafting the manuscript and revising it critically for important intellectual content. All authors have read and approved the final manuscript.
